# Isovitexin targets SIRT3 to prevent steroid-induced osteonecrosis of the femoral head by modulating mitophagy-mediated ferroptosis

**DOI:** 10.1038/s41413-024-00390-0

**Published:** 2025-01-26

**Authors:** Yinuo Fan, Zhiwen Chen, Haixing Wang, Mengyu Jiang, Hongduo Lu, Yangwenxiang Wei, Yunhao Hu, Liang Mo, Yuhao Liu, Chi Zhou, Wei He, Zhenqiu Chen

**Affiliations:** 1 Guangzhou University of Chinese Medicine, Guangzhou, Guangdong China; 2 Musculoskeletal Research Laboratory, Department of Orthopaedics & Traumatology, Li Ka Shing Institute of Health Sciences, The Chinese University of Hong Kong, Hong Kong, China; 3 The Laboratory of Orthopaedics and Traumatology of Lingnan Medical Research Center, Guangzhou University of Chinese Medicine, Guangzhou, China; 4 The Department of Orthopedics, The First Affiliated Hospital of Guangzhou University of Chinese Medicine, Guangzhou, Guangdong China; 5 The Department of Orthopedics, The Third Affiliated Hospital of Guangzhou University of Chinese Medicine, Guangzhou, Guangdong China

**Keywords:** Pathogenesis, Diseases

## Abstract

The death of osteoblasts induced by glucocorticoid (GC)-mediated oxidative stress plays a crucial role in the development of steroid-induced osteonecrosis of the femoral head (SIONFH). Improving bone formation driven by osteoblasts has shown promising outcomes in the prognosis of SIONFH. Isovitexin has demonstrated antioxidant properties, but its therapeutic effects on GC-induced oxidative stress and SIONFH remain unexplored. In this study, we analyzed clinical samples obtained from SIONFH patients using proteomic and bioinformatic approaches. We found an imbalance in mitochondrial homeostasis and ferroptosis-induced impairment of osteogenic capacity in SIONFH. Subsequently, we investigated the cause-and-effect relationship between mitochondria and ferroptosis, as well as the regulatory role of mitophagy in maintaining mitochondrial homeostasis and controlling ferroptosis. We then identified the critical involvement of SIRT3 in modulating mitochondrial homeostasis and ferroptosis. Furthermore, molecular docking and co-immunoprecipitation confirmed the strong interaction between SIRT3 and BNIP3. Strikingly, restoring SIRT3 expression significantly inhibited pathological mitophagy mediated by the BNIP3/NIX pathway. Additionally, we discovered that Isovitexin, by promoting SIRT3 expression, effectively regulated mitophagy, preserved mitochondrial homeostasis in osteoblasts, suppressed ferroptosis, and restored osteogenic capacity, leading to remarkable improvements in SIONFH. These findings reveal the effects and molecular mechanisms of Isovitexin on SIONFH and highlight the potential of targeting SIRT3 as a promising strategy to suppress mitophagy-mediated ferroptosis in osteoblasts and against SIONFH.

## Introduction

Steroid-induced osteonecrosis of the femoral head (SIONFH) stands as the primary manifestation of non-traumatic necrosis in this region, primarily attributed to the excessive and prolonged administration of glucocorticoids (GCs).^1,2^ Without proper intervention, SIONFH demonstrates an alarming collapse rate that exceeds 80% within a 2-year period, leading to a substantial increase in disability rates. Hence, early intervention assumes paramount importance in mitigating the detrimental outcomes associated with SIONFH.^3^ However, the precise underlying mechanism of GC-induced ONFH remains partially elucidated, thus presenting a challenge in the development of targeted therapeutic strategies to effectively halt the progression of SIONFH.^4^

The excessive administration of GCs, which induces osteoblast death, is believed to play a crucial role in the development and progression of SIONFH.^5^ Numerous studies have demonstrated that GCs can trigger osteoblastic death through various pathways, including autophagy, ferroptosis, and apoptosis.^6–13^ As research advances, the role of GCs-induced oxidative stress in influencing osteoblastic death has become increasingly apparent.^6,7,14,15^ Recent investigations have revealed that GCs can stimulate the production of reactive oxygen species (ROS), disrupting the delicate balance between oxidants and antioxidants in the body.^14^ ROS, being highly reactive molecules, have the capability to oxidize lipids, proteins, and DNA, leading to cellular damage.^16^ The excessive generation of ROS overwhelms the endogenous antioxidant defense mechanisms, resulting in a state of oxidative stress and subsequent osteoblastic death.^13,14^ Further exploration of the intricate relationship between GCs-induced oxidative stress and osteoblastic death is crucial for advancing our understanding of SIONFH pathogenesis and developing effective therapeutic interventions.

Mitochondria serve as a critical link connecting oxidative stress and osteoblastic death. While mitochondria are responsible for generating ROS, a significant contributor to oxidative stress, they also possess intrinsic antioxidant systems to counterbalance ROS and maintain cellular redox equilibrium.^17,18^ However, prolonged exposure to oxidative stress compromises mitochondrial homeostasis, impairing their capacity to sustain adequate antioxidant defenses.^19^ Consequently, an excessive release of ROS occurs, further amplifying cellular oxidative stress and establishing a detrimental cycle.^18,19^ Additionally, mitochondria play a vital role in regulating cell death signaling pathways and protein expression, making mitochondrial damage capable of activating various cell death pathways such as mitophagy and ferroptosis.^20,21^ Several studies have explored the relationship between GCs-induced oxidative stress, mitochondrial dysfunction, and subsequent osteoblastic death. These investigations have examined how GCs induce oxidative stress, leading to mitochondrial impairment and ultimately resulting in osteoblastic death.^7,22,23^ Therefore, targeting mitochondria may hold the key to mitigating osteoblastic death triggered by GCs-induced oxidative stress.

Isovitexin, a natural flavonoid compound, possesses remarkable antioxidant and anti-inflammatory properties, making it a valuable regulator in the context of oxidative stress.^24^ Moreover, research studies have highlighted the potential of Isovitexin in modulating osteoblast differentiation through its regulatory effects on mitochondria.^25,26^ Considering the pivotal involvement of oxidative stress and mitochondria in osteoblastic death, Isovitexin emerges as a promising drug for addressing SIONFH.

In this study, utilizing proteomics, we elucidated the mechanisms underlying GCs-induced ONFH, with a particular focus on oxidative stress, mitochondria, and ferroptosis. Subsequently, through in vitro and in vivo experiments, we validated several key findings: 1) confirming the relationship between mitochondria and ferroptosis in the context of oxidative stress, resolving a previously debated aspect^27^; 2) validating the role of SIRT3 in restoring mitochondrial homeostasis through the regulation of mitophagy, thereby demonstrating its capacity to inhibit GCs-induced osteoblastic ferroptosis^28–30^; 3) providing evidence for the potential of Isovitexin in preventing SIONFH through its modulation of SIRT3.

## Results

### There is a disruption of mitochondrial homeostasis and ferroptosis-induced impairment of osteogenic capacity in SIONFH

**Fig. 1:**
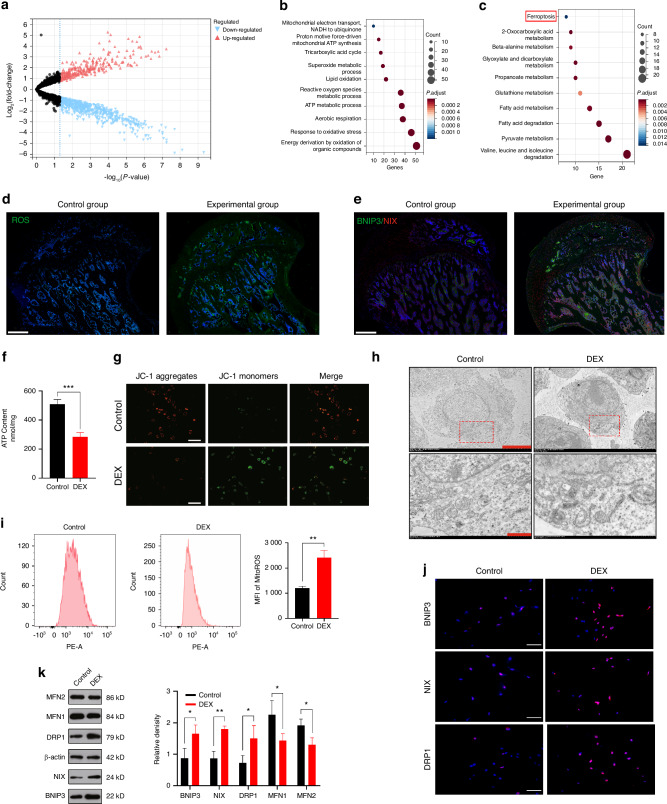
There is a disruption of mitochondrial homeostasis in SIONFH. **a** A volcano plot illustrating the differentially expressed proteins between bone slices obtained from the healthy and necrotic regions of the femoral heads of SIONFH patients. **b** The GO and **c** KEGG enrichment analysis. **d** ROS fluorescent staining and **e** IF double staining of BNIP3/NIX were performed in each group of rat femoral head. Scale bar = 500 μm. **f** Measurement of ATP content in MC3T3-E1 cells following DEX intervention. **g** The evaluation MMP was performed using JC-1 staining following DEX intervention. Scale bar = 200 μm. **h** Assessment of mitophagy levels using TEM after DEX intervention. For regular images, scale bar = 50 μm, and for magnified images, scale bar = 10 μm. **i** Flow cytometry analysis of ROS using mean fluorescence intensity (MFI) in DEX-treated MC3T3-E1 cells. **j** IF staining of BNIP3, NIX, and DRP1 expression in MC3T3-E1 cells treated with DEX. Scale bar = 50 μm. **k** WB analysis of BNIP3, NIX, DRP1, MFN1, and MFN2 expression in MC3T3-E1 cells treated with DEX. In the DEX group, 1 μmol/L DEX was added on the basis of the Control group. The animal sample size is *n* = 5, the cell sample size is *n* = 3. Data were shown as mean ± SD. Student’s *t*-test was used to compare parameters between two groups. *P < 0.05, **P < 0.01, ***P < 0.001, ****P < 0.000 1

To investigate the underlying mechanism of SIONFH, we conducted proteomic analysis on bone slices obtained from the necrotic and healthy regions of the femoral heads of SIONFH patients. The resulting data underwent comprehensive bioinformatics analysis. Initially, differential protein analysis was performed to identify upregulated or downregulated proteins (Fig. 1a and Fig. S1A). Subsequently, GO enrichment and KEGG enrichment analyses were conducted. The GO enrichment analysis revealed significant involvement of biological processes related to mitochondria and oxidative stress (Fig.1b). In the KEGG enrichment analysis, particular attention was given to the ferroptosis pathway due to its intricate association with oxidative stress and mitochondria (Fig. 1c and Fig. S1B).

**Fig. 2:**
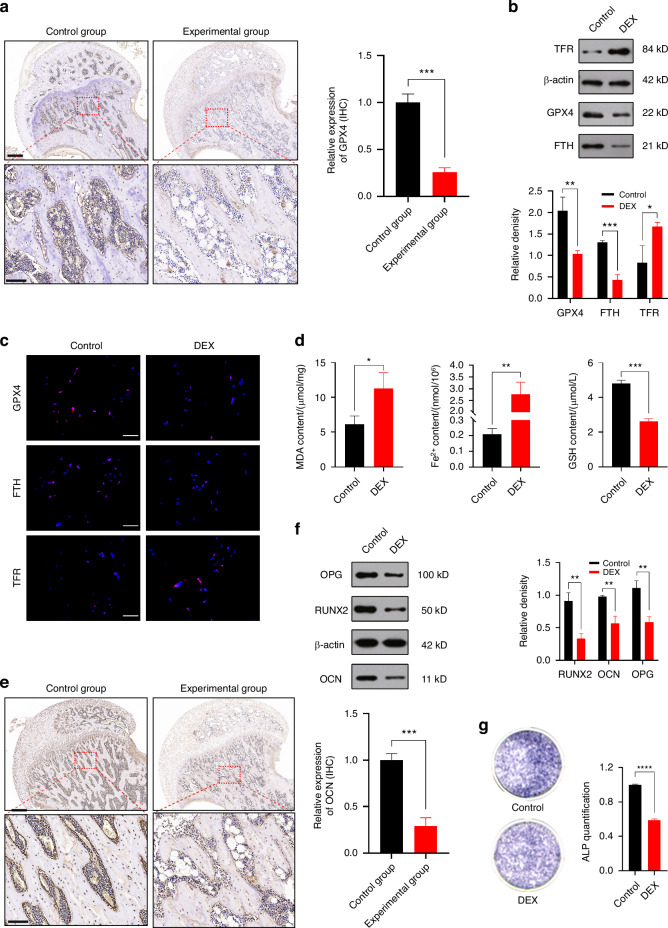
There is ferroptosis-induced impairment of osteogenic capacity in SIONFH. **a** IHC staining of GPX4 expression in each group of rat femoral head. For regular images, scale bar = 500 μm, and for magnified images, scale bar = 100 μm. **b** WB analysis and **c** IF staining of GPX, FTH and TFR expression in MC3T3-E1 cells treated with DEX. Scale bar = 50 μm. **d** Measurement of MDA, Fe^2+^, and GSH content in MC3T3-E1 cells following DEX intervention. **e** IHC staining of OCN expression in each group of rat femoral head. For regular images, scale bar = 500 μm, and for magnified images, scale bar = 100 μm. **f** WB analysis of RUNX, OCN, and OPG expression in the MC3T3-E1 cells treated with DEX. **g** ALP staining in the MC3T3-E1 cells treated with DEX. The animal sample size is *n* = *5*, the cell sample size is *n* = *3*. Data were shown as mean ± SD. Student’s *t*-test was used to compare parameters between two groups. *P < 0.05, **P < 0.01, ***P < 0.001, ****P < 0.000 1

To validate these findings, we then conducted in vivo and in vitro experiments. In the vivo experiment, we established a SIONFH rat model to verify the observations, while in the vitro experiment involved the intervention of GCs on osteoblasts using MC3T3-E1 cells. For the investigation of GCs’ impact on osteoblasts, MC3T3-E1 cells were treated with dexamethasone (DEX). Due to the high dosage typically used in the treatment process, it is necessary for us to select an appropriate concentration of DEX. Based on the result of CCK-8 assay, a concentration of 1 μmol/L was selected for the in vitro experiments, focusing on the consequences of high-dose DEX exposure (Fig. S1C). IF staining of the rat femoral heads revealed a significant increase in ROS levels and pathological elevation of mitophagy-related markers BNIP3/NIX (Fig. 1d, e). Following DEX intervention in MC3T3-E1 cells, a reduction in ATP release (Fig. 1f) and loss of MMP (Fig. 1g) were observed, indicating mitochondrial damage. TEM further confirmed the presence of mitophagy in MC3T3-E1 cell mitochondria following DEX intervention (Fig. 1h and Fig. S1D). Flow cytometry analysis showed elevated levels of ROS, measured by mean fluorescent intensity (MFI), upon DEX treatment (Fig. 1i). Additionally, markers of mitophagy (BNIP3/NIX), fission (DRP1), and fusion (MFN1/MFN2) were examined through IF analysis (Fig. 1j) and WB analysis (Fig. 1k). The results indicated enhanced mitophagy and fission, while mitochondrial fusion was reduced. Furthermore, as the PINK1/PARKIN pathway is also a crucial pathway in mitophagy, we validated the extent of changes in both pathways under DEX intervention through qRT-PCR. The results demonstrated a significant increase in BNIP3/NIX expression (Fig. S1E), while PINK1/PARKIN showed only a slight elevation (Fig. S1F). Therefore, we have shifted the focus of subsequent experiments related to mitophagy towards the BNIP3/NIX pathway. Additionally, IHC staining revealed a significant decrease in GPX4 expression in the femoral heads of SIONFH rats (Fig. 2a). In vitro WB (Fig. 2b), IF staining (Fig. 2c), measurement of MDA levels, Fe^2+^ levels, and GSH levels (Fig. 2d) further confirmed the presence of ferroptosis. Finally, IHC staining for OCN indicated a weakened osteogenic capacity within the femoral heads of SIONFH rats (Fig. 2e). In vitro experiments including qRT-PCR (Fig. S1G), WB analysis (Fig. 2f), and ALP staining (Fig. 2g) provided additional support for the diminished osteogenic capacity.

### Restoring mitochondrial homeostasis can rescue the osteogenic capacity and prevent ferroptosis in MC3T3-E1 cells

Simultaneous occurrence of mitochondrial damage and ferroptosis following DEX intervention raises questions about their relationship. To investigate this, we focused on the role of ROS in maintaining mitochondrial homeostasis and aimed to restore it using a mitochondria-targeted ROS scavenger called XJB-5-131. Subsequently, we assessed the effects of this restoration on ferroptosis and osteoblast differentiation.

**Fig. 3:**
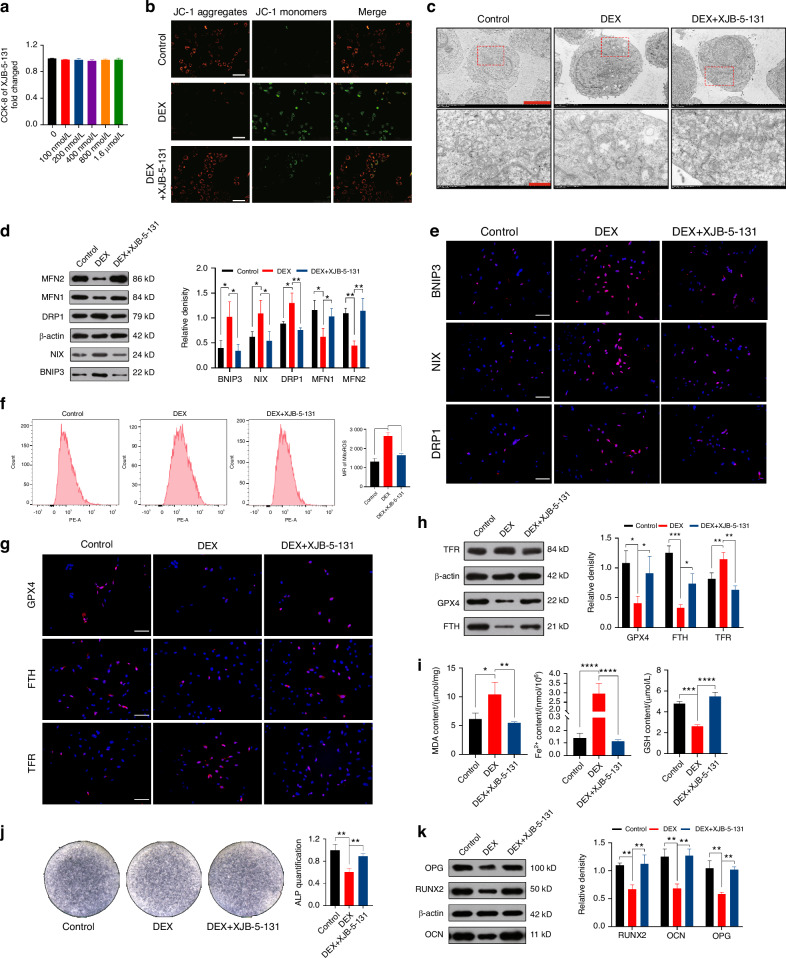
Restoring mitochondrial homeostasis can rescue the osteogenic capacity and prevent ferroptosis in MC3T3-E1 cells. **a** Effect of XJB-5-131 on the proliferation of MC3T3-E1 cells was determined by CCK-8 assay after 2 days of stimulation. **b** The evaluation MMP was performed using JC-1 staining following DEX and XJB-5-131 intervention. Scale bar = 200 μm. **c** Assessment of mitophagy levels using TEM after DEX and XJB-5-131 intervention. For regular images, scale bar = 50 μm, and for magnified images, scale bar = 10 μm. **d** WB analysis of BNIP3, NIX, DRP1, MFN1 and MFN2 expression in MC3T3-E1 cells treated with DEX and XJB-5-131. **e** IF staining of BNIP3, NIX and DRP1 expression in MC3T3-E1 cells treated with DEX and XJB-5-131. Scale bar = 50 μm. **f** Flow cytometry analysis of ROS using MFI after DEX and XJB-5-131 intervention. **g** IF staining and **h** WB analysis of GPX, FTH, and TFR expression in MC3T3-E1 cells treated with DEX and XJB-5-131. Scale bar = 50 μm. **i** Measurement of MDA, Fe^2+^, and GSH content in MC3T3-E1 cells following DEX and XJB-5-131 intervention. **j** ALP staining and **k** WB analysis of RUNX, OCN, and OPG expression in the MC3T3-E1 cells treated with DEX and XJB-5-131. In the DEX + XJB-5-131 group, 1 μmol/L DEX and 800 nmol/L were added. The cell sample size is *n* = *3*. Data were shown as mean ± SD. One-way ANOVA with Bonferroni multiple comparisons test was used for multiple comparisons. *P < 0.05, **P < 0.01, ***P < 0.001, ****P < 0.000 1

To begin, we determined the appropriate intervention concentration of XJB-5-131 and divided the cells into three groups: Control, DEX, and DEX + XJB-5-131. The toxicity of XJB-5-131 on MC3T3-E1 cells was evaluated using a CCK-8 assay (Fig. 3a), and the intervention concentration was determined to be 800 nmol/L. Our initial focus was on the restorative effects of XJB-5-131 on mitochondrial homeostasis. Through JC-1 staining (Fig. 3b), ATP detection (Fig. S1H), and flow cytometry analysis of ROS (Fig. 3f), we observed that XJB-5-131 intervention resulted in the recovery of MMP, increased ATP release, and decreased ROS levels in MC3T3-E1 cells. Furthermore, we employed TEM to examine mitochondrial morphology (Fig. 3c and Fig. S1I) and conducted quantitative analysis of mitophagy, fission, and fusion markers using WB (Fig. 3d) and IF staining (Fig. 3e). The findings indicated that compared to the DEX group, XJB-5-131 treatment reduced mitophagy and fission while promoting mitochondrial fusion. To verify whether restoring mitochondrial homeostasis inhibited ferroptosis, we performed IF staining (Fig. 3g) and WB analysis (Fig. 3h), and measured levels of MDA, Fe^2+^ and reduced GSH (Fig. 3I). These measurements and analyses suggested that mitochondrial regulation plays a crucial role in the occurrence of ferroptosis. Finally, we examined the restorative effects of XJB-5-131 on osteogenic differentiation through ALP staining (Fig. 3j), WB (Fig. 3k), and qRT-PCR (Fig. S1J). The results revealed that the osteogenic capacity was restored in the DEX + XJB-5-131 group compared to the DEX group.

### Mitophagy plays a key role in maintaining mitochondrial homeostasis in MC3T3-E1 cells

To further investigate the role of mitophagy in the regulation of mitochondrial homeostasis following DEX-induced imbalance, we explored whether excessive mitophagy contributes to this imbalance in MC3T3-E1 cells. To inhibit excessive mitophagy, we utilized the mitophagy inhibitor Mdivi-1 and examined whether restoring mitochondrial homeostasis is possible.

**Fig. 4:**
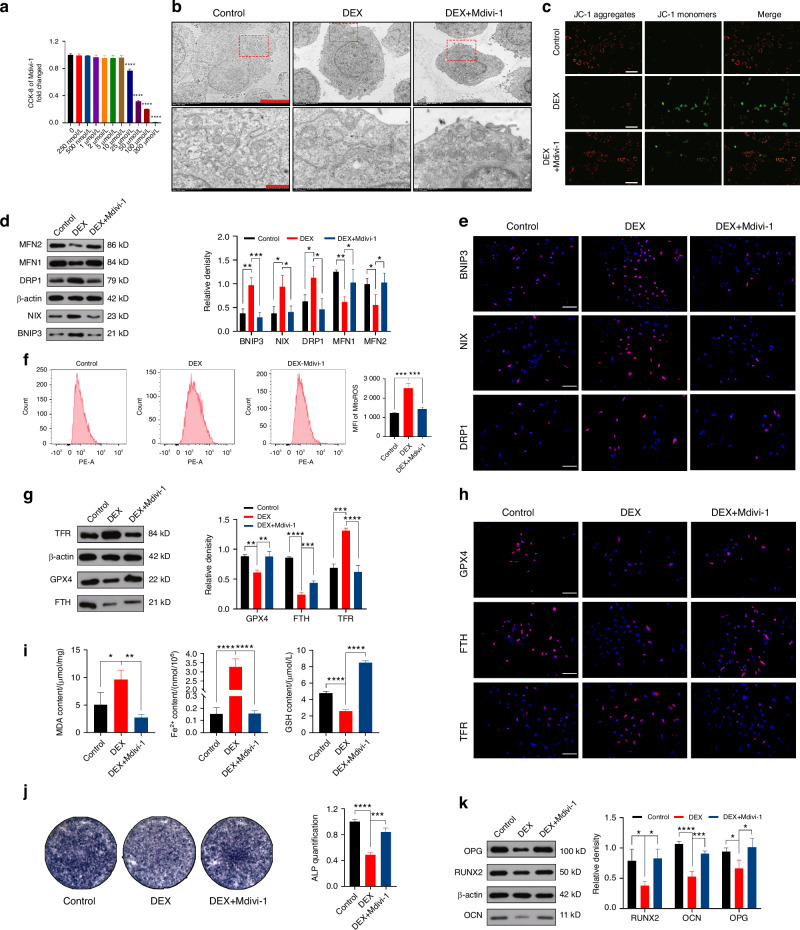
Mitophagy plays a key role in maintaining mitochondrial homeostasis in MC3T3-E1 cells. **a** Effect of Mdivi-1 on the proliferation of MC3T3-E1 cells was determined by CCK-8 assay after 2 days of stimulation. **b** Assessment of mitophagy levels using TEM after DEX and Mdivi-1 intervention. For regular images, scale bar = 50 μm, and for magnified images, scale bar = 10 μm. **c** The evaluation MMP was performed using JC-1 staining following DEX and Mdivi-1 intervention. Scale bar = 200 μm. **d** WB analysis of BNIP3, NIX, DRP1, MFN1 and MFN2 expression in MC3T3-E1 cells treated with DEX and Mdivi-1. **e** IF staining of BNIP3, NIX, and DRP1 expression in MC3T3-E1 cells treated with DEX and Mdivi-1. Scale bar = 50 μm. **f** Flow cytometry analysis of ROS using MFI after DEX and Mdivi-1 intervention. **g** WB analysis and **h** IF staining of GPX, FTH, and TFR expression in MC3T3-E1 cells treated with DEX and Mdivi-1. Scale bar = 50 μm. **i** Measurement of MDA, Fe^2+^, and GSH content in MC3T3-E1 cells following DEX and Mdivi-1 intervention. **j** ALP staining and **k** WB analysis of RUNX, OCN, and OPG expression in the MC3T3-E1 cells treated with DEX and Mdivi-1. In the DEX+ Mdivi-1 group, 1 μmol/L DEX and 5 μmol/L Mdivi-1 were added. The cell sample size is *n* = *3*. Data were shown as mean ± SD. One-way ANOVA with Bonferroni multiple comparisons test was used for multiple comparisons. *P < 0.05, **P < 0.01, ***P < 0.001, ****P < 0.000 1

After determining the safe concentration of Mdivi-1 using a CCK-8 assay, we selected 5 μmol/L as the intervention concentration (Fig. 4a). Initially, using TEM (Fig. 4b and Fig. S1K), we observed that DEX-induced mitophagy was suppressed after Mdivi-1 intervention. WB (Fig. 4d) and IF staining (Fig. 4e) results also indicated the inhibition of mitophagy, along with a decrease in mitochondrial fission and an increase in mitochondrial fusion, compared to the DEX group. The restoration of MMP (Fig. 4c), increased ATP release (Fig. S1L), and reduced ROS levels (Fig. 4f) further confirmed the crucial role of mitophagy in regulating mitochondrial homeostasis. Subsequently, we validated the inhibitory effect of Mdivi-1 on ferroptosis. WB analysis (Fig. 4g) and IF staining (Fig. 4h), along with measurements of MDA, Fe^2+^, and GSH levels (Fig. 4i), indicated that the occurrence of ferroptosis is fundamentally regulated by mitophagy. Finally, we examined the restorative effects of Mdivi-1 on osteogenic differentiation through ALP staining (Fig. 4j), WB (Fig. 4k), and qRT-PCR analysis (Fig. S1M). The results revealed that the osteogenic capacity was restored in the DEX+Mdivi-1 group compared to the DEX group. These findings indicate that the occurrence of ferroptosis is dependent on mitophagy, which plays a vital role in maintaining mitochondrial stability. Inhibition of excessive mitophagy can protect MC3T3-E1 cells from ferroptosis and restore their ability to promote bone formation.

### SIRT3 exerts its regulatory role by modulating mitophagy

**Fig. 5:**
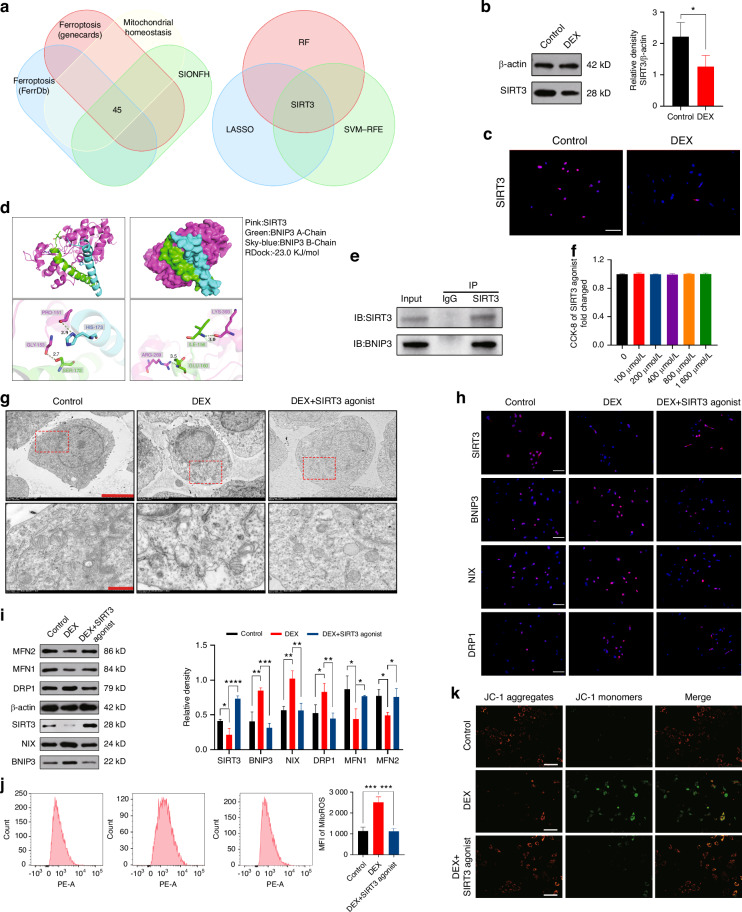
SIRT3 exerts its regulatory role by modulating mitophagy. **a** Firstly, ferroptosis and mitochondria-related proteins were selected from public databases and intersected with the differentially expressed proteins obtained from bone slices of SIONFH patients. A total of 45 proteins were identified from patient samples. Finally, the key protein SIRT3 was determined using Lasso, random forest (RF), and support vector machine with recursive feature elimination (SVM-RFE) methods. **b** WB analysis of SIRT3 expression in MC3T3-E1 cells treated with DEX. **c** IF staining of SIRT3 expression in MC3T3-E1 cells treated with DEX. Scale bar = 50 μm. **d** Molecular docking was performed to predict the interaction between SIRT3 and BNIP3. **e** Co-IP was performed to investigate the interaction between SIRT3 and BNIP3. **f** Effect of SIRT3 agonist on the proliferation of MC3T3-E1 cells was determined by CCK-8 assay after 2 days of stimulation. **g** Assessment of mitophagy levels using TEM after DEX and SIRT3 agonist intervention. For regular images, scale bar = 50 μm, and for magnified images, scale bar = 10 μm. **h** IF staining of SIRT3, BNIP3, NIX and DRP1 expression in MC3T3-E1 cells treated with DEX and SIRT3 agonist. Scale bar = 50 μm. **i** WB analysis of SIRT3, BNIP3, NIX, DRP1, MFN1 and MFN2 expression in MC3T3-E1 cells treated with DEX and SIRT3 agonist. **j** Flow cytometry analysis of ROS using MFI after DEX and SIRT3 agonist intervention. **k** The evaluation MMP was performed using JC-1 staining following DEX and SIRT3 agonist intervention. Scale bar = 200 μm. In the DEX + SIRT3 agonist group, 1 μmol/L DEX and 800 μmol/L SIRT3 agonist was added. The cell sample size is *n* = 3. Data were shown as mean ± SD. One-way ANOVA with Bonferroni multiple comparisons test was used for multiple comparisons. *P < 0.05, **P < 0.01, ***P < 0.001, ****P < 0.000 1

We first selected ferroptosis and mitochondria-related proteins from public data, intersected them with the differentially expressed proteins obtained from bone slices of SIONFH patients, resulting in a total of 45 proteins, all originating from patient samples. Finally, we identified the key protein SIRT3 through three machine-learning techniques (Fig. 5a and Fig. S2A). SIRT3 was found to be downregulated in proteomics analysis, and further confirmation of decreased SIRT3 expression after GCs intervention was provided by WB (Fig. 5b) and IF staining analysis (Fig. 5c). Considering that XJB-5-131 (Fig. S2B, C) and Mdivi-1 (Fig. S2D, E) did not have a regulatory effect on SIRT3, it led us to hypothesize that SIRT3 acts upstream of mitophagy. Given the crucial role of mitophagy in maintaining mitochondrial homeostasis, we postulated that SIRT3 might exert its effects by regulating mitophagy. To investigate this, we conducted molecular docking between SIRT3 and BNIP3, revealing a strong binding affinity between these two proteins (Fig. 5d). Subsequently, we performed Co-IP experiments to further confirm the interaction between SIRT3 and BNIP3 (Fig. 5e). Since DEX treatment resulted in the suppression of SIRT3 expression, we aimed to validate our hypothesis by utilizing a SIRT3 agonist.

**Fig. 6:**
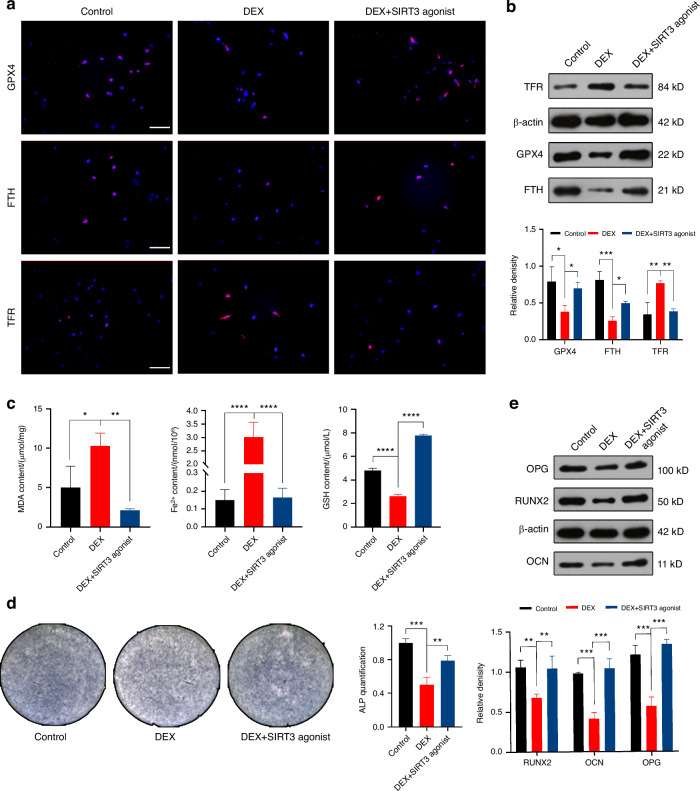
SIRT3 inhibits ferroptosis and restores osteogenic capacity by regulating mitophagy. **a** IF staining and **b** WB analysis of GPX, FTH and TFR expression in MC3T3-E1 cells treated with DEX and SIRT3 agonist. Scale bar = 50 μm. **c** Measurement of MDA, Fe^2+^, and GSH content in MC3T3-E1 cells following DEX and SIRT3 agonist intervention. **d** ALP staining and **e** WB analysis of RUNX, OCN, and OPG expression in the MC3T3-E1 cells treated with DEX and SIRT3 agonist. In the DEX + SIRT3 agonist group, 1 μmol/L DEX and 800 μmol/L SIRT3 agonist were added. The cell sample size is *n* = *3*. Data were shown as mean ± SD. One-way ANOVA with Bonferroni multiple comparisons test was used for multiple comparisons. *P < 0.05, **P < 0.01, ***P < 0.001, ****P < 0.000 1

After determining the safe concentration of the SIRT3 agonist through preliminary experiments, we proceeded with the experiments using a concentration of 800 μmol/L (Fig. 5f). Initially, we validated the regulation of mitophagy by the SIRT3 agonist using TEM (Fig. 5g and Fig. S2F), IF staining (Fig. 5h), and WB (Fig. 5i). The results indicated that the SIRT3 agonist increased SIRT3 expression, inhibited mitophagy and mitochondrial fission, and promoted mitochondrial fusion. Flow cytometry analysis of ROS (Fig. 5j), measurements of ATP levels (Fig. S2G), and JC-1 staining (Fig. 5k) demonstrated that the SIRT3 agonist repaired DEX-induced mitochondrial functional damage. Subsequently, we verified the inhibitory effect of the SIRT3 agonist on ferroptosis (Fig. 6a–c) and its role in restoring osteogenic differentiation (Fig. 6d, e and Fig. S2H).

Collectively, the results from these experiments support the conclusion that SIRT3 restores mitochondrial homeostasis by regulating mitophagy, thereby inhibiting ferroptosis in MC3T3-E1 cells and promoting osteogenic differentiation

### Isovitexin restores GCs-induced mitochondrial damage by upregulating SIRT3

**Fig. 7:**
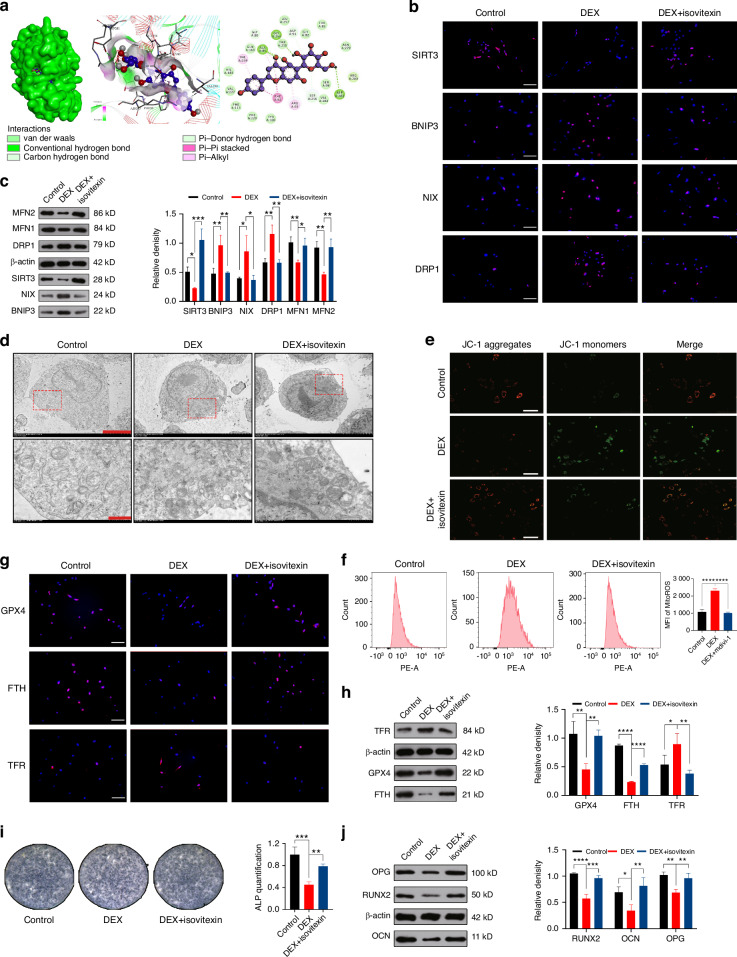
Isovitexin restores GCs-induced mitochondrial damage by upregulating SIRT3. **a** Molecular docking was performed to predict the interaction between Isovitexin and SIRT3. **b** IF staining of SIRT3, BNIP3, NIX and DRP1 expression in MC3T3-E1 cells treated with DEX and Isovitexin. Scale bar = 50 μm. **c** WB analysis of SIRT3, BNIP3, NIX, DRP1, MFN1, and MFN2 expression in MC3T3-E1 cells treated with DEX and Isovitexin. **d** Assessment of mitophagy levels using TEM after DEX and Isovitexin intervention. For regular images, scale bar = 50 μm, and for magnified images, scale bar = 10 μm. **e** The evaluation MMP was performed using JC-1 staining following DEX and Isovitexin intervention. Scale bar = 200 μm. **f** Flow cytometry analysis of ROS using MFI after DEX and Isovitexin intervention. **g** IF staining and **h** WB analysis of GPX, FTH, and TFR expression in MC3T3-E1 cells treated with DEX and Isovitexin. Scale bar = 50 μm. **i** ALP staining and **j** WB analysis of RUNX, OCN, and OPG expression in the MC3T3-E1 cells treated with DEX and Isovitexin. In the DEX+ Isovitexin group, 1 μmol/L DEX and 150 μmol/L Isovitexin was added. The cell sample size is *n* = 3. Data were shown as mean ± SD. One-way ANOVA with Bonferroni multiple comparisons test was used for multiple comparisons. *P < 0.05, **P < 0.01, ***P < 0.001, ****P < 0.000 1

In the final step of the in vitro experiments, we performed molecular docking (Fig. 7a) of Isovitexin with SIRT3 and conducted network pharmacology analysis (Fig. S3A) to investigate the association between Isovitexin and SIONFH. The results demonstrated that Isovitexin not only exhibited strong binding affinity (−8.1 kcal/mol) with SIRT3 but also primarily targeted oxidative stress and mitochondria in relation to SIONFH. We determined the safe concentration of Isovitexin to be 150 μmol/L and proceeded with the validation of its regulatory effect on SIRT3 (Fig. S3B). Through IF staining (Fig. 7b), WB (Fig. 7c), and TEM (Fig. 7d and Fig. S3D), we confirmed that Isovitexin increased the expression of SIRT3, promoted mitochondrial fusion, and inhibited mitophagy and fission by activating SIRT3. Further assessments using JC-1 staining (Fig. 7e), measurements of ATP levels (Fig. S3C), and flow cytometry analysis of ROS (Fig. 7f) clarified the restorative effect of Isovitexin on mitochondrial function. Similar to the previous experiments, we also confirmed the inhibitory effect of Isovitexin on ferroptosis (Fig. 7g, h and Fig. S3E) and its ability to restore osteogenic differentiation (Fig. 7i, j and Fig. S3F).

To enhance the reliability and reproducibility of the study, we further validated using rat BMSCs. We added three key experiments involving osteogenesis, mitochondrial function, and ferroptosis. qRT-PCR results indicated an increase in RUNX2 expression after drugs intervention (Fig. S4B), while JC-1 staining (Fig. S4A) and MDA detection (Fig. S4C) also confirmed results similar to the aforementioned experiments.

The results of this part of the experiment confirmed that Isovitexin restores GCs-induced mitochondrial damage and prevents ferroptosis by upregulating SIRT3 in MC3T3-E1 cells. Isovitexin activates SIRT3, leading to the promotion of mitochondrial fusion, inhibition of mitophagy and fission, and restoration of mitochondrial function.

### Isovitexin targets SIRT3 to prevent SIONFH by modulating mitophagy-mediated ferroptosis

In order to further validate the conclusions drawn from the cell experiments, animal experiments were conducted. The animals were divided into six groups: Control group, Experimental group, XJB-5-131 group, Mdivi-1 group, SIRT3-agonist group, and Isovitexin group, based on the grouping used in the in vivo experiments.

**Fig. 8:**
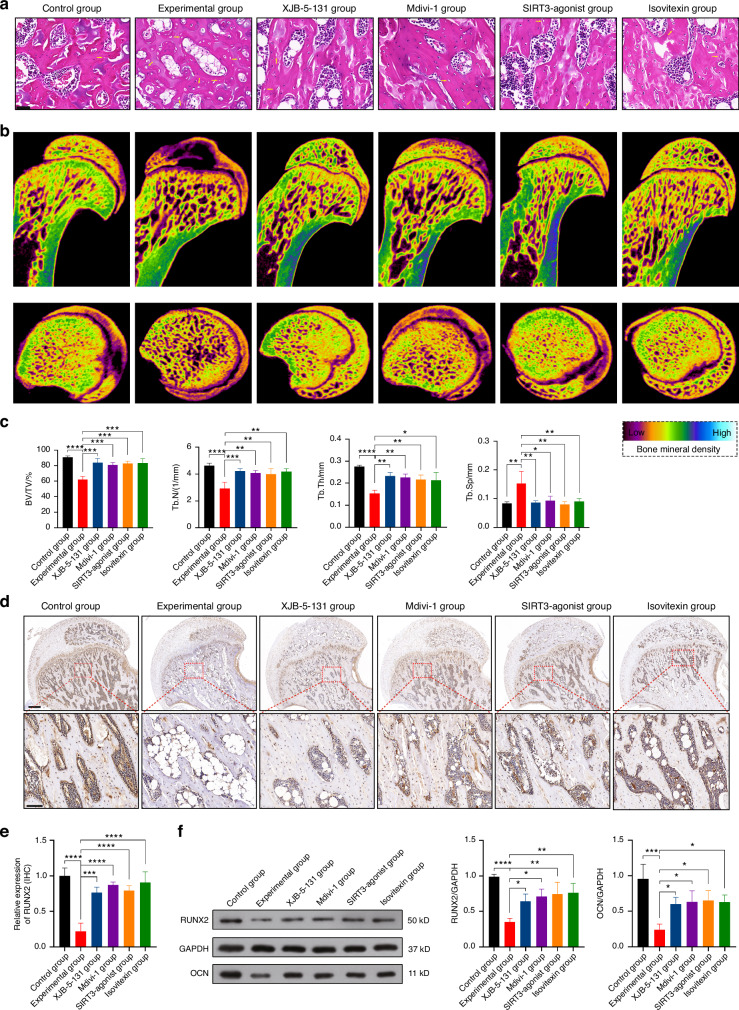
Isovitexin against SIONFH by promoting osteogenesis. **a** HE staining was performed to assess the success of the model induction (The yellow arrow indicates empty lacunae). Scale bar = 50 μm. **b** After Micro CT scanning, two-dimensional imaging of the microstructure of the femoral head in each group of rats was obtained. Blue-white color represents high bone density, while red-purple color represents low bone density. **c** The bone volume fraction (BV/TV), trabecular thickness (Tb.Th), trabecular number (Tb.N), and trabecular spacing (Tb.Sp) of the femoral head in each group of rats were measured (*n* = 5). **d** and **e** IHC staining of RUNX2 expression in each group of rats (*n* = 5). For regular images, scale bar = 500 μm, and for magnified images, scale bar = 100 μm. **f** WB analysis of RUNX2 and OCN expression in each group of rats (*n* = 3). Data were shown as mean ± SD. One-way ANOVA with Bonferroni multiple comparisons test was used for multiple comparisons. *P < 0.05, **P < 0.01, ***P < 0.001, ****P < 0.000 1

First, HE staining was performed to assess the success of the model induction. The results showed increased empty lacunae in the Experimental group, indicating the presence of SIONFH. However, the drug groups exhibited lower empty lacunae, indicating the therapeutic effect of the drugs on SIONFH rats (Fig. 8a). Micro CT results revealed that BV/TV, Tb.N, and Tb.Th were the lowest in the Experimental group, but the drug groups showed significant improvements in these parameters. On the other hand, Tb.Sp was higher in the drug groups compared to the Experimental group, which was consistent with the expected results (Fig. 8b, c).

Next, the expression of osteogenic markers in each group was analyzed using IHC staining (Fig. 8d, e) and WB (Fig. 8f). The results showed that although the expression of RUNX2 and OCN was not as high as in the Control group, they were significantly increased compared to the Experimental group in the drug groups.

**Fig. 9:**
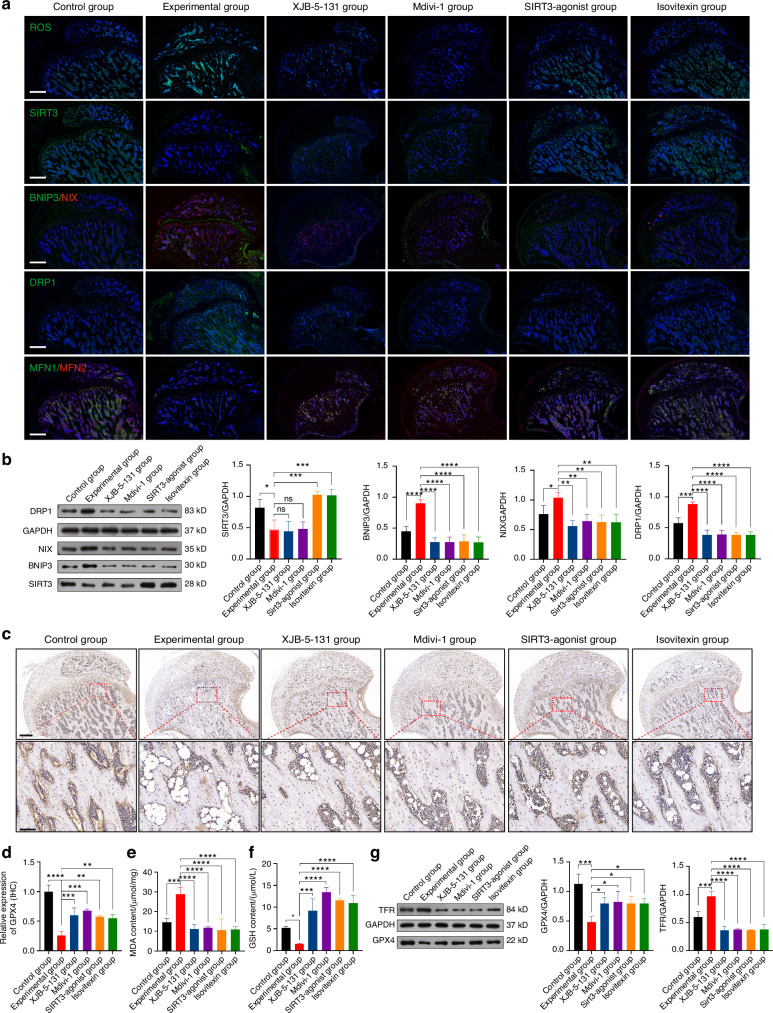
Isovitexin targets SIRT3 to prevent SIONFH by modulating mitophagy-mediated ferroptosis. **a** ROS fluorescent staining, IF single staining of SIRT3 and DRP1, and IF double staining of BNIP3/NIX and MFN1/MFN2 were performed in each group of rats. Scale bar = 500 μm. **b** WB analysis of SIRT3, BNIP3, NIX and DRP1 expression in each group of rats (*n* = 3). **c**, **d** IHC staining of RUNX2 expression in each group of rats (*n* = 5). For regular images, scale bar = 500 μm, and for magnified images, scale bar = 100 μm. **e** Measurement of MDA and **f** GSH content were performed in each group of rats (*n* = 5). **g** WB analysis of GPX4 and TFR expression in each group of rats (*n* = 3). Data were shown as mean ± SD. One-way ANOVA with Bonferroni multiple comparisons test was used for multiple comparisons. *P < 0.05, **P < 0.01, ***P < 0.001, ****P < 0.000 1

Furthermore, to validate the mechanisms mentioned in the in vitro experiments, mitochondrial-related indicators were measured. IF staining showed a reduction in ROS expression in all four drug groups (Fig. 9a). Further results from IF staining and WB analysis revealed that the expression of SIRT3 in the XJB-5-131 and Mdivi-1 groups remained unchanged compared to the Experimental group, while it was increased in the SIRT3-agonist and Isovitexin groups, consistent with the results of the in vitro experiments (Fig. 9a, b). IF staining and WB analysis also examined the expression of key markers such as BNIP3, NIX, MFN1, MFN2, and DRP1. The results showed that mitophagy and fission were enhanced, while fusion was reduced in the Experimental group. However, the four drug groups were able to reverse this condition and restore mitochondrial homeostasis (Fig. 9a, b).

Finally, indicators related to ferroptosis were observed. The results from IHC staining (Fig. 9c, d), MDA detection (Fig. 9e), GSH detection (Fig. 9f), and WB analysis (Fig. 9g) showed that ferroptosis occurred significantly in the Experimental group, while the drug groups effectively inhibited ferroptosis.

In conclusion, based on this part of the animal experiment, it can be ultimately concluded that Isovitexin targets SIRT3 to prevent SIONFH by modulating mitophagy to inhibit osteoblastic ferroptosis. The results support the potential of Isovitexin in preventing SIONFH.

## Discussion

While the exact pathogenesis of SIONFH remains unclear, it is evident that enhancing bone repair significantly improves prognosis.^31,32^ The process of bone repair relies on maintaining a delicate balance between osteoblast-mediated bone formation and osteoclast-mediated bone resorption.^33^ However, the presence of GCs triggers osteoblast apoptosis, resulting in suppressed bone formation.^34^ This insufficient bone formation creates a “mechanically weak zone” within the femoral head, thereby increasing the risk of femoral head collapse.^35^ Consequently, investigating the underlying causes of osteoblastic death is crucial for comprehending the pathogenic mechanisms of SIONFH and exploring effective prevention and treatment modalities.

To initiate our study, we conducted proteomic analysis on both necrotic and healthy regions of the femoral head in SIONFH patients, followed by bioinformatics analysis. Our findings shed light on the pivotal role of oxidative stress, mitochondrial dysfunction, and ferroptosis in the development of SIONFH. Additionally, we discovered that mitophagy plays a crucial regulatory role in mitochondrial homeostasis and ferroptosis. Subsequently, we conducted in vitro and in vivo experiments to validate the regulatory influence of mitochondria on ferroptosis in the presence of GCs. Progressing further, we identified SIRT3 as a key target protein and demonstrated SIRT3 can restore mitochondrial function by modulating mitophagy, effectively inhibiting ferroptosis in osteoblasts. Finally, we successfully demonstrated the potential of Isovitexin in preventing SIONFH by enhancing SIRT3 expression.

Mitochondria, which are double-membrane organelles, play crucial roles in energy production, cellular metabolism, and the regulation of cell death processes.^36^ Ferroptosis is a regulated form of cell death characterized by uncontrolled lipid peroxidation and is highly dependent on iron levels.^37^ The ROS generated by mitochondria during oxidative stress play a crucial role in the occurrence of ferroptosis.^27^ Additionally, the notable morphological features observed under electron microscopy in cells undergoing ferroptosis, such as condensed mitochondria and increased membrane density, are closely associated with mitochondrial alterations.^38^ Therefore, there is a belief in the causal relationship between mitochondria and ferroptosis. Indeed, there are studies presenting contrary viewpoints. Jelinek et al. observed that although mitochondrial function was restored in both a mouse model and cells undergoing ferroptosis induction after using the mitochondrial ROS scavenger MitoQ, the expression of GPX4 continued to decrease, and lipid peroxidation levels increased.^39^ Furthermore, Gao et al. demonstrated that mitochondria play a crucial role in ferroptosis induced by cysteine depletion, but they did not have a significant impact on ferroptosis induced by inhibiting GPX4.^27^ Given the ongoing debate surrounding the role of mitochondria in regulating ferroptosis, the mitochondrial-targeted antioxidant XJB-5-131 was employed to investigate their relationship. Our results demonstrated a restoration of mitochondrial parameters to their normal state upon treatment with XJB-5-131, accompanied by a substantial suppression of ferroptosis. These findings strongly support the involvement of mitochondria in the regulation of this intricate process.

Mitophagy, also known as mitochondrial clearance or mitochondrial selective autophagy, is a crucial cellular process that plays a vital role in regulating mitochondrial homeostasis.^40,41^ It not only eliminates damaged mitochondria, preventing the release of harmful substances and the initiation of cell death signals, but also controls the quantity and quality of mitochondria to maintain an appropriate cellular level. This process is essential for preserving cellular energy metabolism, balancing oxidative stress, and ensuring cell survival.^40–42^ In fact, an increasing number of studies have begun to focus on the relationship between mitophagy and ferroptosis.^43–45^ Li et al. found that mitophagy, mediated by both BNIP3 and PINK1 pathways, plays a protective role against cisplatin-induced ferroptosis in renal tubular epithelial cell through the regulation of the ROS/HO1/GPX4 axis.^46^ Rademaker G discovered that mitophagy can increase labile iron pools by releasing iron from numerous iron-sulfur clusters involved in oxidative phosphorylation. The exacerbation of mitophagy will elevate the lysosomal iron content of mitochondrial iron-sulfur clusters, leading to lysosomal iron leakage and resulting in ferroptosis.^47^ Yu et al. proposed that the enhanced mitochondrial fragmentation and mitophagy release the iron pool within mitochondria, providing a significant additional source of labile iron, thereby rendering cells more susceptible to ferroptosis.^48^ Our study findings also indicate that the restoration of mitochondrial homeostasis and the inhibition of ferroptosis were achieved upon treatment with the mitophagy inhibitor Mdivi-1. This observation suggests that the presence of ferroptosis is dependent on mitophagy in SIONFH.

SIRT3, residing within the mitochondrial inner membrane, stands as the exclusive enzyme endowed with stable mitochondrial deacetylase activity. It assumes a pivotal role in the deacetylation of diverse proteins at distinct mitochondrial sites, thereby orchestrating the regulation of mitochondrial homeostasis.^49^ By modulating proteins within cellular organelles, SIRT3 governs crucial aspects such as oxidative stress, mitophagy, ATP generation, and energy metabolism.^50–52^ Notably, owing to its remarkable antioxidant capabilities, SIRT3 has been postulated as a significant target in the battle against cellular ferroptosis.^29,53^ Our proteomic study has unveiled the pivotal role of SIRT3 in the pathological progression of SIONFH. Furthermore, following treatment with XJB-5-131 and Mdivi-1, the expression of SIRT3 remains unaltered, underscoring its upstream regulatory role in mitophagy. Co-IP experiments further elucidated the interaction between SIRT3 and the classical mitophagy marker BNIP3. Subsequent treatment with a SIRT3 agonist restored mitophagy and suppressed ferroptosis. These findings unequivocally demonstrate that SIRT3 mitigates ferroptosis by controlling mitophagy through the regulation of BNIP3 expression.

Isovitexin has attracted significant attention in our research due to its outstanding anti-inflammatory and antioxidant properties.^24^ Tao et al. have shown that Isovitexin activates the SKN-1/Nrf2 pathway, leading to the upregulation of antioxidant genes and proteins, thereby reducing ROS accumulation and contributing to the delay of aging.^54^ The research by Hao R revealed that Isovitexin can alleviate skin aging and damage by inhibiting Nrf2/Keap1/HO-1-related oxidative damage and LC3II/p62/GATA4-related autophagy.^55^ Pal et al.’s study has further intrigued us by demonstrating that Isovitexin, through AdipoRs and the mitochondrial biogenesis pathway PGC-1α, induces oxidative phosphorylation and ATP synthesis, resulting in osteoblast differentiation.^25^ Additionally, another study from Pal suggests that Isovitexin has significant oral bioavailability and can be translated into bone anabolic effects comparable to teriparatide and inhibits bone resorption.^56^ Based on the aforementioned literature, our bioinformatics analysis, and molecular docking studies, we speculate that Isovitexin may exert its osteogenesis by modulating oxidative stress and mitochondrial homeostasis. Consequently, we conducted in vitro and in vivo experiments with Isovitexin. Our findings indicate that Isovitexin can enhance antioxidant capabilities by upregulating SIRT3 to regulate mitophagy, consequently restoring mitochondrial function.

This study also presents certain limitations. Firstly, it exclusively used male rats as research subjects, although SIONFH can potentially affect both males and females. Therefore, future research should consider including both genders in the study scope. Secondly, unlike in adult humans, we observed that the growth plate in adult rat femoral heads remains open. It remains uncertain whether this difference plays a role in the progression of SIONFH. Although significant alterations in the early growth plate of SIONFH rats were not detected in this study, this issue requires further comprehensive investigation. Lastly, a key characteristic of SIONFH is vascular damage. However, this study primarily focused on bone repair and did not quantify vascular changes in femoral head histology. Therefore, future research directions could explore whether interventions aimed at bone repair also affect vascular repair within the femoral head.

**Fig. 10:**
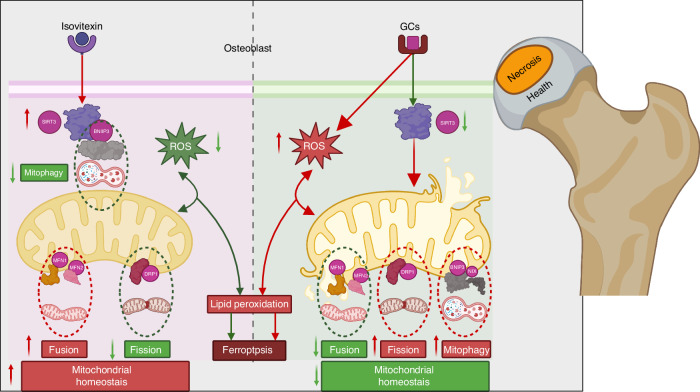
Isovitexin targets SIRT3 to prevent steroid-induced osteonecrosis of the femoral head by modulating mitophagy-mediated ferroptosis

Taken together, our study demonstrated that the effects of Isovitexin on SIONFH are dependent on SIRT3. Briefly, Isovitexin upregulates SIRT3, inhibits excessive mitophagy, and restores the mitochondrial homeostasis imbalance caused by oxidative stress induced by GCs, thereby reducing ferroptosis in osteoblasts. In conclusion, our study reveals the significant role of SIRT3 in regulating mitophagy and maintaining mitochondrial homeostasis (Fig.10). Additionally, our findings emphasize the promising approach of targeting SIRT3 to inhibit mitophagy-mediated ferroptosis in osteoblasts to against SIONFH.

## Materials and methods

### Collection of femoral head specimens

In this study, we conducted our research using a cohort of patients with SIONFH who underwent total hip arthroplasty at the Department of Orthopedics of the First Affiliated Hospital of Guangzhou University of Chinese Medicine. The study was conducted following the approval of the Ethical Committee of the First Affiliated Hospital of Guangzhou University of Chinese Medicine (Ethics No. K-2023-181). The selection of patients adhered to specific inclusion criteria outlined as follows: a) Patients were diagnosed with SIONFH based on the Chinese Adult Femoral Head Necrosis Clinical Diagnosis and Treatment Guidelines (2020). b) Early stages of SIONFH such as Association Research Circulation Osseous (ARCO) stages II and IIIA. c) Patients had a clear history of glucocorticoid drug use, with no evidence of hip trauma or long-term alcohol abuse. d) Patients fell within the age range of 18 to 55 years. e) Patients were free from other bone metabolic diseases. f) Prior to participation, patients were fully informed about the study and provided their consent to participate. Nine patients who met these inclusion criteria were recruited for the study. Following total hip arthroplasty, tissue samples of the femoral head were collected. The femoral head tissue was then cut into uniformly sized bone slices, approximately 5 mm thick, along the coronal surface. These bone slices were subsequently stored in an ultra-low temperature refrigerator at −80 °C for future use in the experiments.

### Proteomics and bioinformatics analysis

Protein data-independent acquisition (DIA) analysis has proven to be effective and reliable, allowing for a comprehensive exploration of the proteome while preserving relevant data. In this study, we focused on analyzing healthy and necrotic regions within the examined specimens using protein-DIA techniques. Our objective was to uncover unique expression patterns of proteins in different anatomical areas. The raw data obtained were pre-processed and imported into R statistical software for further analysis and visualization. Gene ontology (GO) function and KEGG pathway enrichment analyses of genes corresponding to significantly different proteins among groups were performed using the “clusterProfiler” package in R (v.4.3.1). Additionally, we obtained the corresponding protein structures in PDB format from the Protein Crystal Structure Database (PDB, https://www.rcsb.org). To investigate the interaction between drugs and target proteins, we conducted molecular docking using RDock, a widely used docking software. The binding energy was used as a measure to evaluate the effectiveness of the docking process, with higher binding energy indicating better binding activity of the drug to the receptor protein and a more stable docking state.

### Animal experiments

A total of 60 male SD rats, 10 weeks old and of SPF-grade, were randomly divided into six groups: Control group, Experimental group, XJB-5-131 group, Midiv-1 group, SIRT3-agonist group, and Isovitexin group, with 10 rats in each group. Except for the Control group, lipopolysaccharide (2 mg/kg, Sigma) was injected into the tail vein of rats in the other groups. On days 2, 3, and 4, rats in the experimental groups received methylprednisolone injections (30 mg/kg, MedChemExpress) into the gluteal muscle, alternating between the two gluteal muscles. After the completion of the modeling process, the XJB-5-131 (a mitochondria-targeted ROS scavenger, MedChemExpress), Midiv-1 (a mitophagy inhibitor, MedChemExpress), and Isovitexin (Chengdu, Must) groups received intraperitoneal injections of 1 mg/kg,^57^ 15 mg/kg,^47^ and 15 mg/kg,^58^ respectively, every two days. The SIRT3-agonist group received a daily oral gavage of 200 mg/kg SIRT3 agonist^59^ (Nicotinamide riboside chloride, MedChemExpress). The Control group was injected with an equal amount of 0.9% saline. After 4 weeks from the modeling process, all rats were euthanized, and bilateral femurs were collected for further analysis. The animal study was conducted following the approval of the Ethical Review Committee for Animal Experimentation of Guangzhou University of Chinese Medicine (Ethics No. 20230526001).

### Micro-computed-tomography scanning

The proximal femur was scanned using a SkyScan micro-CT scanner, with the femoral head as the central area. The scanner was set with basic parameters, including a ray intensity of 1 mm Al, a resolution of 9 μmol/L, a voltage of 65 kV, a current of 385 μA, and a scanning length of 18 mm. Following the scanning process, the original image data were reconstructed using NRecon software. The reconstructed model was then adjusted in three dimensions using Data Viewer software to ensure accurate alignment and positioning. To perform a quantitative analysis of the model, CTAn software was used. This software provided metrics for specific indicators, including bone volume fraction (BV/TV), trabecular number (Tb.N), trabecular thickness (Tb.Th), and trabecular separation (Tb.Sp) of the bone tissue. These indicators offer insights into the density and microarchitecture of the trabecular bone in the proximal femur. Furthermore, Database Viewer software was utilized to reconstruct two-dimensional (2D) images based on the analyzed data. This allowed for visualizing and examining the bone structure in a simplified format.

### Hematoxylin and eosin (HE) staining

The left lower limb femurs of rats from each group were taken and fixed in 4% paraformaldehyde for 48 h and then decalcified using 14% EDTA (PH = 7.4). After the completion of decalcification, the tissues were dehydrated, paraffin-embedded, sectioned (thickness of 5 μm) and baked, and then stained in a series of operations in a fume hood using Harris Hematoxylin Stain and Eosin Stain. The sections were routinely dehydrated, clarified and sealed, then the proximal femoral region was scanned using a tissue section scanner. The number of empty bone lacunae and the ratio of empty bone lacunae versus total bone lacunae were compared within each group.

### Immunohistochemistry (IHC) staining

The bone tissue paraffin sections were deparaffinized with distilled water, followed by antigen retrieval and blocking. Subsequently, the RUNX2 (ABways) and GPX4 (Proteintech) antibodies was added dropwise onto the sections, which were then incubated at 4 °C overnight. After washing with water, secondary antibody was added and incubated for 30 min at 37 °C. Finally, SABC was added and incubated for 30 min at 37 °C, and DAB staining was performed for 5–10 min. The sections were routinely dehydrated, clarified, and sealed, then photographed under observation, and the rate of immunohistochemistry positivity was counted in each field of view.

### Immunofluorescence (IF) staining and ROS staining

Paraffin sections of bone tissue were dewaxed and subjected to antigen retrieval treatment. Sections were permeabilized with 0.3% TritonX-100, blocked with 5% goat serum for 2 h. Drops were added with the SIRT3 (Proteintech), BNIP3 (ABways), NIX (ABways), MFN1 (Proteintech), MFN2 (Proteintech) and DRP1 (Abcam) antibody or ROS stain solution and incubated overnight at 4 °C. After washing with PBST, the secondary antibody was added, and finally, anti-fluorescence quencher containing DAPI for mounting was used. Fluorescence microscopy was detected to observe the fluorescence signal in the femoral head region and the images were analyzed using lmage J.

### Cell culture and cellular interventions

The resuscitated MC3T3-E1 cells were spread on a 100 mm dish containing α-MEM medium with 10% fetal bovine serum by volume, and incubated at 37 °C in an incubator with 5% CO_2_ by volume. The medium was changed every 2 days, and the cells were passaged when the cell fusion was about 90%. Stably grown MC3T3-E1 cells were differentiated into osteoblasts by culturing in osteogenic induction solution (complete α-MEM medium containing 10 nmol/L dexamethasone, 50 μg/mL vitamin C and 5 mmol/L β-glycerophosphate). Stably grown MC3T3-E1 cells were taken, digested, centrifuged, resuspended, and spread, and the cells were grouped and intervened after the cells were attached to the wall. The cells were divided into the following six groups: Control group, DEX (Dexamethasone, MedChemExpress) group, XJB-5-131 group, Midiv-1 group, SIRT3-agonist group, Isovitexin group. The intervention program was finalized by reviewing the relevant literature and pre-experiments. Control group: α-MEM complete medium containing osteoinductive fluid; DEX group: α-MEM complete medium containing osteoinductive fluid +1 μmol/L DEX; DEX + XJB-5-131 group, DEX+Midiv-1 group, DEX + SIRT3-agonist group, and DEX+Isovitexin group: on the basis of the DEX group, respectively add 800 nmol/L XJB-5-131, 800 μmol/L SIRT3 agonist, 5 μmol/L Midiv-1, 150 μmol/L Isovitexin.

### Isolation and culture of rat bone marrow mesenchymal stem cells (BMSCs)

BMSCs were used to validate the experimental results of MC3T3-E1. SD rats were anesthetized and euthanized by decapitation. The tibia and fibula were dissected to expose the bone marrow cavity, which was then flushed with PBS to collect the bone marrow fluid. The collected bone marrow fluid was cultured in α-MEM medium supplemented with 10% fetal bovine serum, 1% penicillin, and streptomycin. The cell culture medium was refreshed every 2 days. When the cell growth reached 80%–90% confluence, the cells were passaged. Cells from the third passage were harvested and utilized in the subsequent experiments.

### Cell proliferation assay

The MC3T3-E1 cells or BMSCs were grown in 96-well plates at a density of 5 000 cells/well, and after intervention treatment, the medium was discarded and washed with PBS. After reintroducing 100 μL of medium and setting up blank control wells, 10 μL was added to each well, and after incubation for 1–2 h at 37 °C and 5% CO_2_, the absorbance (OD value) at 450 nm was measured by an enzyme marker.

### Alkaline phosphatase (ALP) staining

The above stably grown MC3T3-E1 cells were spread in 48-well plates at a density of 6 × 10^3^ cells/well, and the experimental group was induced to differentiate by adding 800 nmol/L XJB-5-131, 800 μmol/L SIRT3 agonist, 5 μmol/L Midiv-1 and 150 μmol/L Isovitexin in osteogenic induction solution per well, respectively; and the negative control group was cultured using α-MEM complete culture. The medium was changed every 2 days, and the cells were cultured for 7 days until osteoblasts were maturely differentiated. After 7 days, the cells were washed once with PBS and fixed using 4% paraformaldehyde for 30 min. Add the reaction substrate 150 μL/well according to the instructions of Alkaline Phosphatase Assay Kit reagent and incubate at room temperature away from light for 12 h. After washing with PBS for three times, the 48-well plate was placed in a ventilated kitchen to air-dry. Finally, the 48-well plates were scanned using a laser scanner and the staining depth of each group of cells was quantified using Image J software.

### Mitochondrial membrane potential (MMP) analysis

Stably grown cells were spread in 6-well plates at a density of 3 × 10^5^ cells/well, and the cells in each group were treated accordingly, rinsed once with PBS, and then 1 mL of cell culture medium was added. Add 1 mL of JC-1 staining solution to each well, and reset to incubate in the incubator for 20 min. Discard the supernatant at the end of staining, and wash the cells with JC-buffer 2 times. Add 2 mL of culture medium and place under fluorescence microscope or laser confocal microscope to observe the results and collect pictures. Observe and detect the fluorescence value by fluorescence microscope and fluorescence spectrophotometer.

### Transmission electron microscopy (TEM) observation

Stably grown MC3T3-E1 cells were spread in 6-well plates at a density of 3 × 10^5^ cells/well, and 2.5% room-temperature glutaraldehyde fixative was added after 24 h of cell intervention in each group. The cells were fixed at room temperature for 5 min, scraped off, and centrifuged for 2 min, and a new electron microscopy fixative was added to suspend the cells. After being put at 4 °C for 30 min, fixed dehydrated sections were made, and mitochondria were observed under transmission electron microscope and photographed.

### Ferrous ion (Fe^2+^), malondialdehyde (MDA), glutathione (GSH) and adenosine triphosphate (ATP) detection

Stably grown MC3T3-E1 cells were spread in 6-well plates at a density of 3 × 10^5^ cells/well. After 24 h of cell intervention in each group, the culture medium was discarded, the cells were collected. and the extraction solution was added according to the instructions of the kit, and after ultrasonic crushing, centrifugation, and taking the supernatant. The absorbance of the supernatant was measured by a spectrophotometric assay at 532 nm for MDA, 405 nm for GSH. Determination of RLU values for ATP using a luminometer.

### Flow cytometry for ROS detection

Stably grown MC3T3-E1 cells were spread in 12-well plates and the cellular intervention is performed as in “2.8”. Prepare MitoSOX Red (5 mmol/L, MedChemExpress) in the ratio of 1:1 000, incubate at 37 °C for 30 min, wash the cells with PBS and collect them in a centrifuge tube, centrifuge at 1 000 r/min for 5 min, discard the supernatant, and resuspend the cells with PBS. The relative content of ROS in each group of samples was measured by flow cytometry.

### Co-immunoprecipitation (Co-IP) assay

After intervention, the cells were lysed, homogenized, and centrifuged and the supernatant was taken, 2.0 ug SIRT3 monoclonal antibody was added and incubated at 4 °C for 1 h. 20 μL protein G agarose was added and incubated inverted at 4 °C for 3–5 h. The cells were then centrifuged at 1 000 *g* for 5 min at 4 °C. The supernatant was discarded, and the immunization mixture was collected, and the precipitate was suspended in the SDS-PAGE upsampling buffer for Western blot experiments.

### Western blotting (WB)

The collected cellular protein samples were separated by SDS-PAGE, transmembrane, closed with TBST blocking solution containing 5% skimmed milk for 1 h. The membrane was washed three times by TBST, and SIRT3, BNIP3 (Cell antibody usage with ABclonal; bone tissue antibody usage with ABways), NIX (Cell antibody usage with Santa Cruz; bone tissue antibody usage with ABways), DRP1, MFN1, MFN2, GPX4(Cell antibody usage with Affinity; bone tissue antibody usage with Proteintech), TFR (Abcam), FTH (Affinity), RUNX2 (Wanleibio), OCN (Wanleibio) and OPG (Wanleibio) antibody was incubated at 4 °C overnight. On the following day, the corresponding secondary antibody was added, incubated at room temperature for 1 h, the membrane was washed by TBST, the color was developed by ECL chemiluminescence, imaging was performed by gel imager, and quantitative analysis was performed using Image J software.

### Quantitative real-time polymerase chain reaction (qRT-PCR)

RNA was extracted from bone tissue powder or cells using the Trizol method, RNA concentration was determined by a biophotometer, and reverse transcription was performed using Oligo-dT and other reagents. Finally, the expression of RUNX2 (Forward: 5′-AGTCCCAACTTCCTGTGCT-3′, Rveverse: 5′- GGTGAAACTCTTGCCTCGTC-3′), OCN (Forward: 5′- GAGGGCAATAAGGTAGTGAA-3′, Rveverse: 5′- CATAGATGCGTTTGTAGGC-3′) and OPG (Forward: 5′-AGGGCGTTACCTGGAGAT-3′, Rveverse: 5′- AGGGTGCTTTCGATGAAG-3′) was detected by SYBR Green method, using Hprt as the housekeeping gene, using Bio-Rad real-time fluorescence PCR instrument, in which the RNA reverse transcription program was 42 °C 60 min, 92 °C 10 min, 4 °C, the PCR reaction on-line program was Step1 95 °C 30 s, Step2 (40 Cycles) 95 °C 15 s, Step2 (40 Cycles) 95 °C 15 s, Step3 95 °C 15 s. ΔΔCt method was used to quantitatively analyze the relative expression of target genes.

### Statistical analysis

SPSS 21.0 software was used to analyze the results of the measurement data, which conformed to normal distribution. One-way ANOVA was used for comparison between multiple groups. Independent samples *t*-test was used for comparison between two groups when the variance was uniform, and nonparametric Mann-Whitney test was used when the variance was not uniform. *P* < 0.05 indicated that the difference was statistically significant.

## Supplementary information

Supplementary figure Legends
