# Supplementary material for: Isovitexin targets SIRT3 to prevent steroid-induced osteonecrosis of the femoral head by modulating mitophagy-mediated ferroptosis
Source: Bone Res. 2025 Jan 26;13:18. doi: 10.1038/s41413-024-00390-0 (PMC11770138; doi:10.1038/s41413-024-00390-0)
Supplement: Supplementary file 1 — Supplementary figure Legends [file 41413_2024_390_MOESM1_ESM.docx]

**Figure S1**


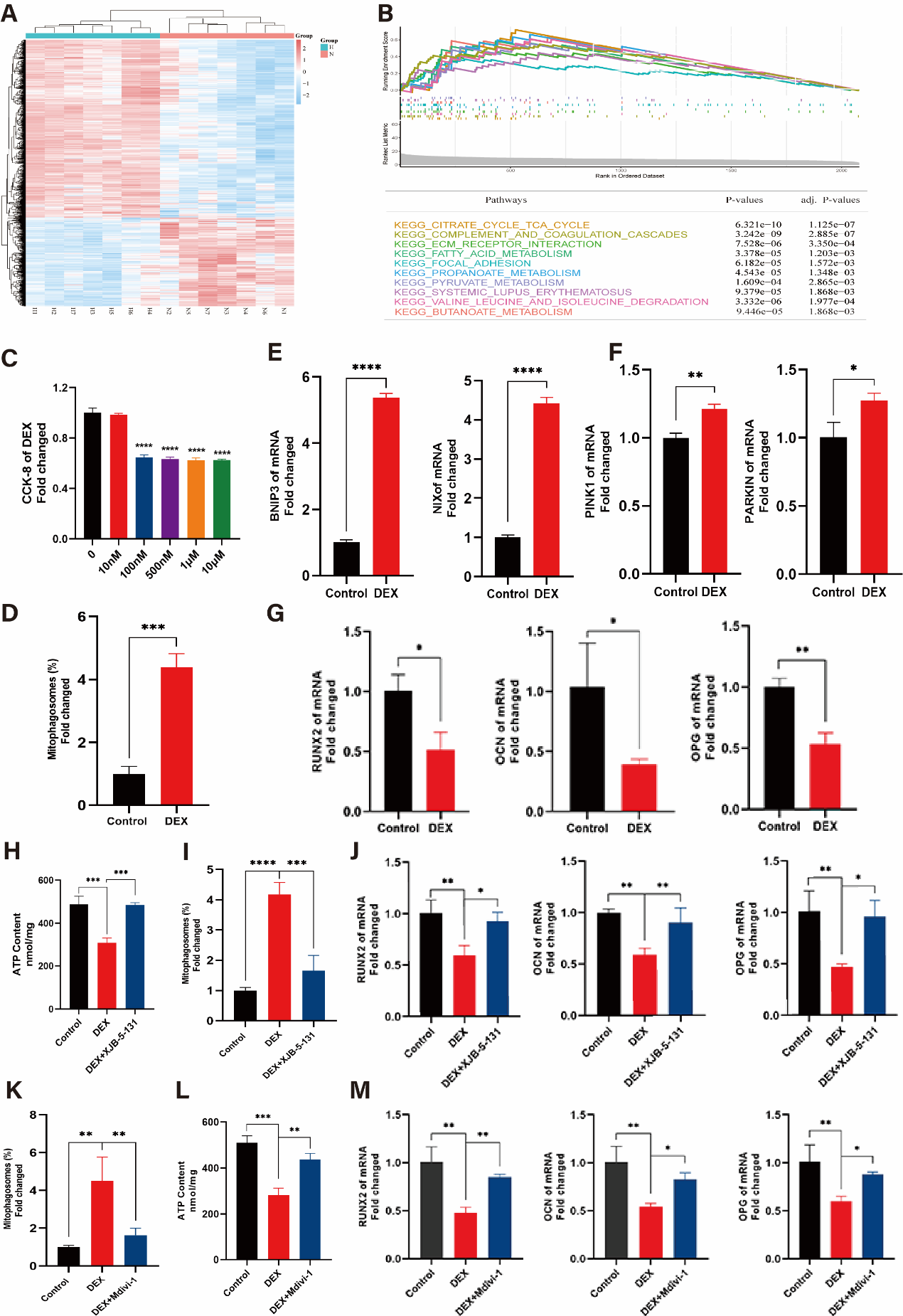


(A) Heatmap illustrating differentially expressed proteins between the healthy and necrotic regions. (B) The KEGG enrichment analysis. (C) Effect of DEX on the proliferation of MC3T3-E1 cells was determined by CCK-8 assay after 2 days of stimulation. (D) The ratio of the number of mitochondrial autophagosomes to the total number of mitochondria in each cell treated with DEX. (E) qRT-PCR analysis of BNIP3 and NIX expression in the MC3T3-E1 cells treated with DEX. (F) qRT-PCR analysis of PINK1 and PARKIN expression in the MC3T3-E1 cells treated with DEX. (G) qRT-PCR analysis of RUNX2, OCN, and OPG expression in the MC3T3-E1 cells treated with DEX intervention. (H) Measurement of ATP content in MC3T3-E1 cells following DEX and XJB-5-131 intervention. (I) The ratio of the number of mitochondrial autophagosomes to the total number of mitochondria in each cell treated with DEX and XJB-5-131. (J) qRT-PCR analysis of RUNX, OCN, and OPG expression in the MC3T3-E1 cells treated with DEX and XJB-5-131. (K) The ratio of the number of mitochondrial autophagosomes to the total number of mitochondria in each cell treated with DEX and Mdivi-1. (L) Measurement of ATP content in MC3T3-E1 cells following DEX and Mdivi-1 intervention. (M) qRT-PCR analysis of RUNX, OCN, and OPG expression in the MC3T3-E1 cells treated with DEX and Mdivi-1. The cell sample size is *n=3*. Data were shown as mean ± SD. One-way ANOVA with Bonferroni multiple comparisons test was used for multiple comparisons. **p* < 0.05, ** *p* < 0.01, *** *p* < 0.001, **** *p* < 0.0001.

**Figure S2**


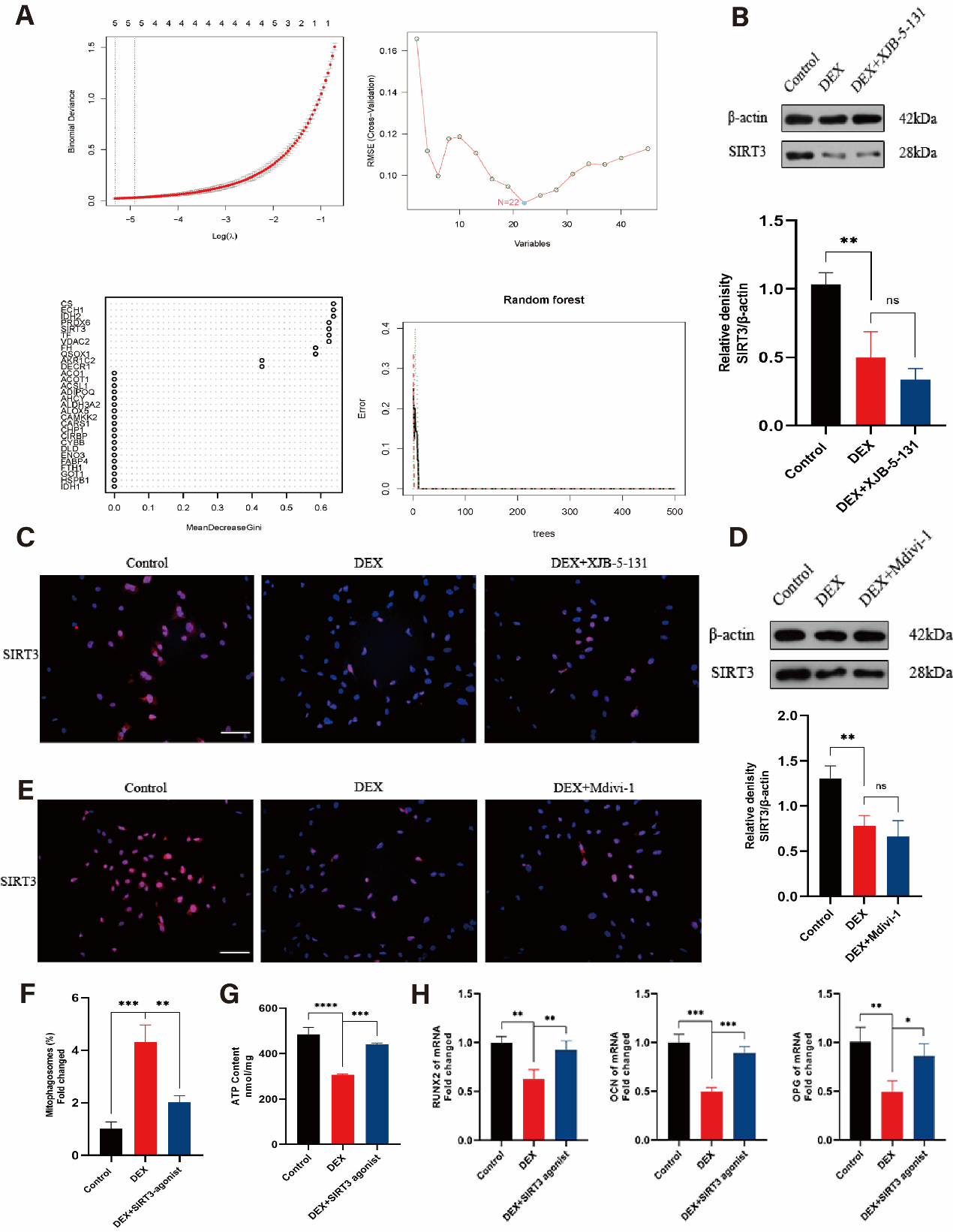


(A) Lasso, random forest (RF), and support vector machine with recursive feature elimination (SVM-RFE) were used for feature selection to identify key target protein SIRT3. (B) WB analysis and (C) IF staining of SIRT3 expression in MC3T3-E1 cells treated with DEX and XJB-5-131. Scale bar = 50 μm. (D) WB analysis and (E) IF staining of SIRT3 expression in MC3T3-E1 cells treated with DEX and Mdivi-1. Scale bar = 50 μm. (F) The ratio of the number of mitochondrial autophagosomes to the total number of mitochondria in each cell treated with DEX and SIRT3 agonist. (G)Measurement of ATP content in MC3T3-E1 cells following DEX and SIRT3 agonist intervention. (H) qRT-PCR analysis of RUNX, OCN, and OPG expression in the MC3T3-E1 cells treated with DEX and SIRT3 agonist. The cell sample size is *n=3*. Data were shown as mean ± SD. One-way ANOVA with Bonferroni multiple comparisons test was used for multiple comparisons. **p* < 0.05, ** *p* < 0.01, *** *p* < 0.001, **** *p* < 0.0001.

**Figure S3**


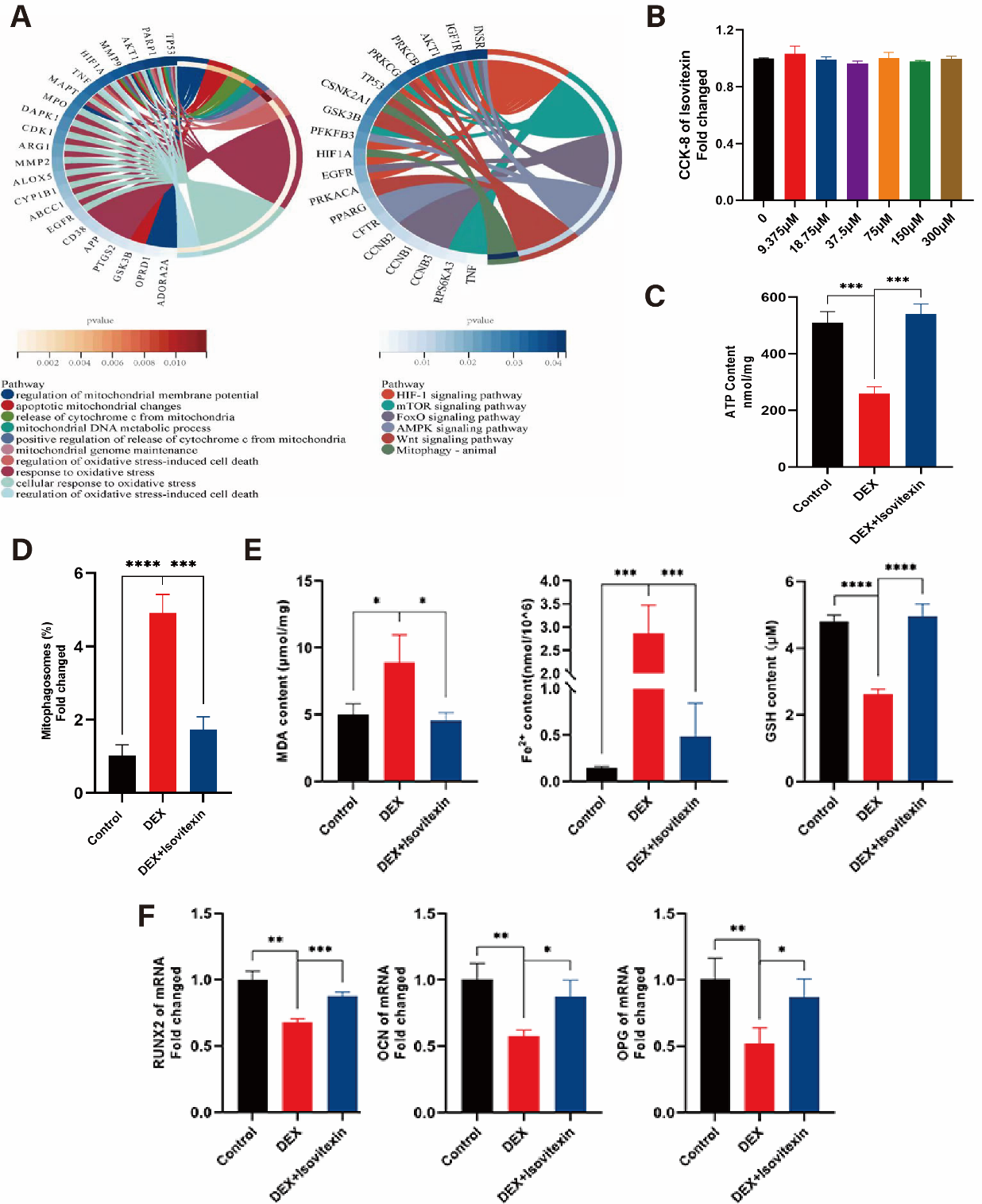


(A) The GO (Left) and KEGG (Right) enrichment analysis of Isovitexin with SIONFH.

(B) Effect of Isovitexin on the proliferation of MC3T3-E1 cells was determined by CCK-8 assay after 2 days of stimulation. (C) Measurement of ATP content in MC3T3-E1 cells following DEX and Isovitexin intervention. (D) The ratio of the number of mitochondrial autophagosomes to the total number of mitochondria in each cell treated with DEX and Isovitexin. (E) Measurement of MDA, Fe^2+^, and GSH content in MC3T3-E1 cells following DEX and Isovitexin intervention. (F) qRT-PCR analysis of RUNX, OCN, and OPG expression in the MC3T3-E1 cells treated with DEX and Isovitexin. In the DEX+ Isovitexin group, 1μM DEX and 150 μM/mL Isovitexin was added. The cell sample size is *n=3*. Data were shown as mean ± SD. One-way ANOVA with Bonferroni multiple comparisons test was used for multiple comparisons. **p* < 0.05, ** *p* < 0.01, *** *p* < 0.001, **** *p* < 0.0001.

**Figure S4**


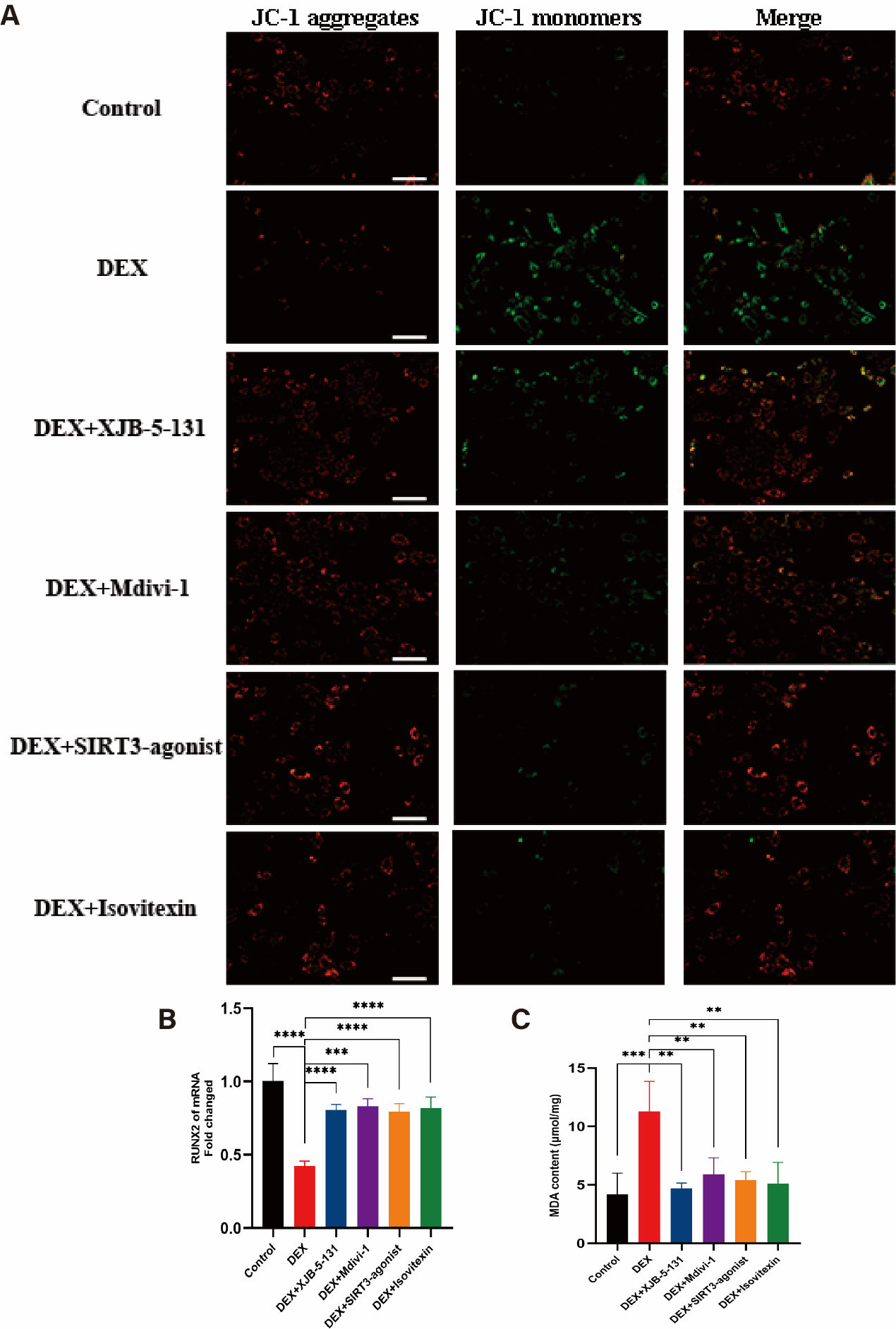


(A) The evaluation MMP was performed using JC-1 staining following DEX, XJB-5-131, Mdivi-1, SIRT3-agonist and Isovitexin intervention. Scale bar = 200 μm. (B) qRT-PCR analysis of RUNX2 expression in the BMSCs treated with DEX, XJB-5-131, Mdivi-1, SIRT3-agonist and Isovitexin intervention. (C) Measurement of MDA in BMSCs following DEX, XJB-5-131, Mdivi-1, SIRT3-agonist and Isovitexin intervention. The cell sample size is *n=3*. Data were shown as mean ± SD. One-way ANOVA with Bonferroni multiple comparisons test was used for multiple comparisons. **p* < 0.05, ** *p* < 0.01, *** *p* < 0.001, **** *p* < 0.0001.
